# Characterization of genomic DNA sequence of the candidate gene for *FB_Mfu10* associated with fire blight resistance in *Malus* species

**DOI:** 10.1186/s13104-021-05709-2

**Published:** 2021-07-27

**Authors:** Ofere Francis Emeriewen, Henryk Flachowsky, Andreas Peil

**Affiliations:** Julius Kühn-Institut (JKI), Federal Research Centre for Cultivated Plants, Institute for Breeding Research On Fruit Crops, Dresden, Germany

**Keywords:** *FB_Mfu10*, MAL0045, Mfu10, *Malus fusca* fire blight resistance locus

## Abstract

**Objective:**

The proposed candidate gene underlying the *Malus fusca* fire blight resistance locus on chromosome 10 was previously predicted to possess 880 amino acids and 8 exons. Eight base pair (8 bp) insertion/deletion in the first exon potentially distinguished resistant genotypes from susceptible ones. This study aimed at analyzing the candidate gene sequence in another set of original resistant and susceptible progeny, characterizing the sequence in a transgenic line transformed with the candidate gene under its own native promoter, as well as deciphering the potential genomic differences between this candidate gene and its homolog in the ‘Golden Delicious’ doubled haploid genome (GDDH13).

**Results:**

Sequences of amplicons of part of the candidate gene amplified in two progenies that showed resistant and susceptible fire blight phenotypes, confirmed the 8 bp insertion that distinguishes susceptible and resistant progenies. The transgenic line was positive for the candidate gene sequence, confirming a successful transfer into the background of apple cultivar ‘Pinova’, and possessed the same genomic sequence as the progeny with a resistant phenotype. Sequence analysis showed that the homolog gene on GDDH13 possesses a significant 18 bp deletion in exon 1 leading to a difference of 15 amino acid from the protein sequence of the candidate gene.

**Supplementary Information:**

The online version contains supplementary material available at 10.1186/s13104-021-05709-2.

## Introduction

Several *Malus* species, especially cultivars of the domesticated apple (*Malus domestica* Borkh.) are susceptible to fire blight disease—caused by the bacterium *Erwinia amylovora* [1–3]. Nevertheless, strong resistance is found in individual accessions of different wild *Malus* species [4]—for example in *M*. ×*robusta* 5 [5, 6], *M. floribunda* 821, ‘Evereste’ [7], *M*. ×*arnoldiana* MAL0004 [8] and *M. fusca* MAL0045 [9]. Only in some of these genotypes have candidate genes been detected, which underlie mapped fire blight resistance loci on different linkage groups [10–13].

The candidate gene proposed to underlie the fire blight resistance locus of *Malus fusca* MAL0045 possesses B-lectin, PAN-apple and serine/threonine kinase domains, 8 exons and 880 amino acids [13]. This candidate gene was predicted on the sequences of bacterial artificial chromosomes (BACs)—46H22/24N24, which spanned the resistance locus on linkage group 10 (LG10) [14]. The donor of this gene, *M. fusca* accession MAL0045, is highly resistant to *E*. *amylovora*, with the resistance locus detected on LG10 following artificial inoculation of MAL0045 × ‘Idared’ progeny with different wild types and mutant strains of the bacterium [15]. One of the apple reference genomes—the ‘Golden Delicious’ doubled haploid (GDDH13) sequence [16], was integral in characterizing the fire blight region on LG10 by aligning sequences of closely linked markers as well as BAC clones in order to characterize the region relative to this apple reference genome [13]. Emeriewen et al. [13] amplified the open reading frame (ORF) of the gene in clones 46H22/24N24 spanning the region for resistance and in clone 94B13, spanning the homolog region, with sequencing of the amplicons detecting an 8 bp insertion present in clone 94B13.

## Main text

To characterize the candidate gene sequence for *FB_Mfu10*, PCR was applied on *M. fusca* (MAL0045), ‘Idared’, and clones 46H22 and 94B13 to prove amplification of part of the ORF (3539 bp) as well as the entire ORF and its border sequences upstream and downstream (6366 bp) as described in [13]. PCR was also applied on two progeny individuals of MAL0045 × ‘Idared’, 05210-147, which displays a resistant phenotype to *E. amylovora* strain Ea222, and 05210-89, which displays a susceptible phenotype to the same strain. Briefly, PCR was performed using the DreamTaq Hot Start PCR Master Mix Kit (Thermo Scientific, Berlin, Germany) in a 12 μl volume with the following conditions: 95 °C for 5 min, followed by 34 cycles of 95 °C for 1 min, 68 °C for 90 s and 72 °C for 6 min, and an extension of 72 °C for 10 min. Table 1 shows the primers used in this study. 20–40 ng of DNA were used for PCR [13]. Purification of PCR products was performed using the MSB Spin PCRapace kit (Invitek GmbH, Berlin, Germany) according to the manufacturer’s protocol. Eurofins MWG Operon (Ebersberg, Germany) conducted sequencing of the amplification products. The resulting sequences were analysed using the National Centre for Biotechnology (NCBI) BLAST® program or by aligning two sequences with each other. Gene prediction analyses with sequences was done using FGENESH with algorithms for dicot Arabidopsis. Predicted proteins were analyzed as described in [13].

**Table 1:** Primers used in this study

Primer name	Forward	Reverse	Size (bp)
*npt*II	ACAAGATGGATTGCACGCAGG	AACTCGTCAAGAAGGCGATAG	780
FB_Mfu10_RSeq1^a^	TTCATATCAGCCCTTTTAACCACTGCTACA	GCCATAATTAAAACCTAGCCACTATCCCGT	6366
MVH_FB_Mfu10^a^	GCTAGCTGCAGAACTTGCTTGCTCA	GAGAAAGAGAAAACCGCCCCGTCT	3539
FR_FB_Mfu10^a^	AAAGCGGATATTTATTGGCACTGGTATCAC	AACGGTGTGCTTTGATTTGTCAACATAGAT	1577
FB_Mfu10-Rseq-ORF^b^	AATTTCATCATGGTATCAGAGTGGGTTGTC	TGTTCTTGTCCAAGGTAAGAACTCCTGTTG	896

^a^Emeriewen et al. [13]

^b^Developed in this study

To obtain a transgenic line, fire blight susceptible apple cultivar ‘Pinova’ was transformed with a construct carrying the candidate gene sequence. This construct was obtained by cloning the ORF and its border sequences into a D034p9-Dao-FLPi_STPK (*FB_Mfu10*) vector (Additional file 1: File S1). Cloning was performed by DNA cloning service (Hamburg, Germany). Agrobacterium-mediated transformation and regeneration were carried out according to the established protocols of Julius Kühn Institute, Dresden-Pillnitz, Germany, as described by Flachowsky et al. [17].

DNA was isolated from desired samples using Qiagen DNeasy Plant Mini Kit (Qiagen, Hilden, Germany) according to the manufacturer’s protocol. The transgenic line was tested with *npt*II primer pair and with candidate gene-specific primers (Table 1). PCR analyses using *npt*II primer pair and FB_Mfu10-Rseq-ORF primer pair were performed in a 20-µl volume consisting of 1 × Dream Taq buffer (ThermoFisher Scientific, Darmstadt, Germany), 0.2 mM dNTPs, 1 µM each of forward and reverse primers, 0.5 U Dream Taq DNA polymerase (ThermoFisher Scientific, Darmstadt, Germany) and 2-µl of DNA (20 ng). Running PCR conditions for *npt*II test were 94 °C for 4 min, followed by 25 cycles of 94 °C for 30 s, 56 °C for 1 min and 72 °C for 1 min and an extension of 72 °C for 7 min. Running PCR conditions for FB_Mfu10-Rseq-ORF were 94 °C for 4 min, followed by 32 cycles of 94 °C for 30 s, 67 °C for 1 min and 72 °C for 1 min and an extension of 72 °C for 5 min. To amplify the ORF in the transgenic line with MVH_FB_Mfu10 primer pair, CloneAmp™ Hifi PCR Premix kit (Takara, Saint-Germain-en-Laye, France) was used according to the manufacturer’s instruction with a running PCR program of 98 °C for 30 s, followed by 34 cycles of 98 °C for 10 s, 65 °C for 15 s and 72 °C for 30 s and an extension of 72 °C for 10 min.

Total RNA was extracted from transgenic line and *M. fusca *in vitro seedling using InviTrap® Spin Plant RNA Mini Kit according to the manufacturer’s protocol (Stratec, Berlin, Germany). DNA contamination removal and cDNA synthesis were performed using the Invitrogen DNA-free™ Kit (Thermo Scientific, Berlin, Germany) and the RevertAid First Strand cDNA Synthesis Kit (Thermo Scientific, Berlin, Germany), respectively, and according to the manufacturers’ protocols. The conditions for amplifying other gene-specific primers are as described earlier [13].

**Fig. 1:**
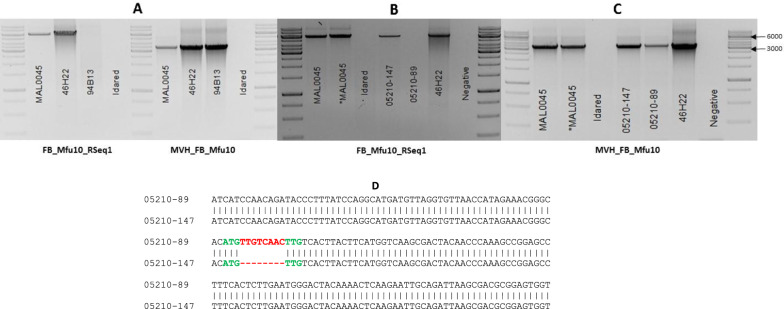
Amplification with primers that amplify the ORF together with the border sequences upstream and downstream—6366 bp (FB_Mfu10_RSeq1) and part of the ORF alone—3539 bp (MVH_FB_Mfu10). 6366 bp fragment is amplified only in resistant MAL0045 and clone 46H22 but the 3539 bp appears to be amplified in both resistant and susceptible clones but not in the susceptible parent— ‘Idared’ (**a**). Similarly, the progeny with a resistant phenotype to Ea222—05210-147 amplifies the 6366 bp but not the progeny with a susceptible phenotype—05210-89 (**b**). Like the susceptible clone, 94B13, 05210-89 appears to amplify the 3539 bp (**c**). Sequencing of amplicons amplified by 05210-147 and 05210-89 revealed the 8 bp insertion (highlighted in red) present in susceptible genotypes (**d**). *MAL0045 = 40 ng of DNA used. 1 kb ladder used

Only in *M. fusca* and clone 46H22 was the ORF together with its border regions (6366 bp) amplified. A fragment of the ORF (~ 3539 bp) was amplified in clone 94B13, whereas the susceptible parent ‘Idared’ failed to amplify any gene specific fragments tested (Fig. 1a). Similarly, the 6366 bp was amplified in the resistant progeny, 05210-147, but not in the susceptible progeny, 05210-89 (Fig. 1b). However, similar ORF fragments were amplified in both progenies (Fig. 1c). Sequencing of amplicons amplified by 46H22 and 94B13 confirmed the 8 bp insertion, which is possessed by the susceptible clone. In parallel, amplicon sequencing confirmed that the susceptible progeny, 05210-89 also possesses this 8 bp insertion but not the resistant progeny, 05210-147 (Fig. 1d).

The obtained sequences from amplicons of the aforementioned samples were aligned against each other and against the predicted sequence of the candidate gene. In addition, the predicted sequence of the candidate gene, 3555 bp in total, was aligned against the GDDH13 genome using BLASTN [18] via the Rosaceae website available at https://www.rosaceae.org/. The output from this alignment showed a best hit of 97% identity (3456/3554 bp) and gaps of 1% (23/3554). The best-hit alignment of these sequences (Additional file 2: File S2) were in the region between 30,708,564 and 30,712,095 on chromosome 10 of the GDDH13 genome [16]. In comparison to the candidate gene sequence, there is a significant 18 bp deletion in the GDDH13 homolog sequence (Additional file 2: File S2). Gene prediction analyses with the homolog sequence from GDDH13 predicted an ORF with the same domains as the candidate gene but with 15 amino acids difference (i.e. 865 aa—Additional file 3: File S3).

**Fig. 2:**
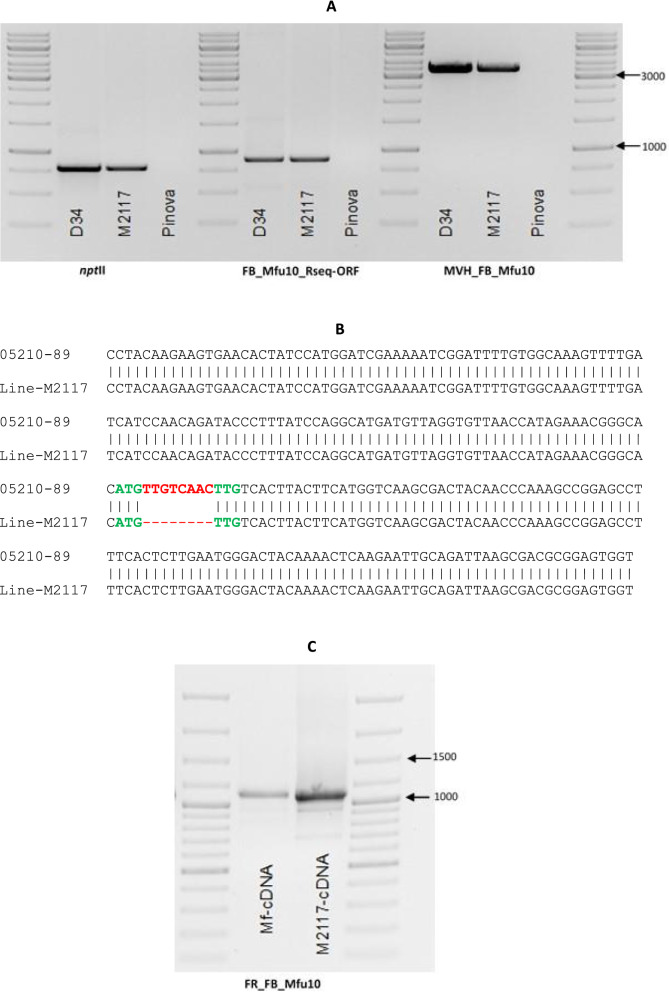
Proof that transgenic line M2117 is positive for the selectable marker gene *npt*II, and gene-specific marker (**a**). FB_Mfu10-RSeq-ORF primer amplifies part of the border sequence and part of the first exon. D34 plasmid DNA (construct plasmid DNA) and ‘Pinova’ serve as positive and negative controls, respectively. 1 kb ladder used. Sequencing of amplicon amplified by line M2117 and compared with the susceptible progeny 05210-89 shows the same characteristics as the sequence as the resistant 05210-147 **(b)** lacking the 8 bp insertion. *M*. *fusca* and M2117 cDNAs amplify only exons sequence—1104 bp using FR_FB_Mfu10 (**c**), which borders a 1577 bp region of the candidate gene

A transgenic line, M2117, was successfully generated following agrobacterium-mediated transformation of apple cultivar ‘Pinova’ with the construct carrying the candidate gene with its border sequences. The correct *npt*II fragment (780 bp) in the construct was amplified in M2117 as well as the candidate gene-specific primer, which amplifies a section of the border sequence upstream and part of the ORF, and the 3539 bp of almost the entire ORF (Fig. 2a). Sequencing of amplicons amplified by M2117 showed the same characteristics of 05210-147 unique to resistant progenies (Fig. 2b). RT-PCR also showed M2117 was positive for the candidate gene (Fig. 2c).

It is quite clear from these results and as initially reported by Emeriewen et al. [13] that 8 bp differentiates fire blight susceptible individuals from resistant individuals within the MAL0045 × ‘Idared’ progeny. However, the failure of the susceptible progeny to amplify the ORF plus border sequences strongly suggests that the border (promoter) regions of this gene might play a key role in fire blight resistance. It was reported that this 8 bp causes a difference of 28 amino acids in the protein sequence, which is potentially sufficient to affect the corresponding phenotype. Nonetheless, we cannot rule out the role of the promoter. The apple cultivar ‘Pinova’ used for genetic engineering in this study is susceptible to fire blight [19]. The transfer of *FB_Mfu10* candidate gene sequence into ‘Pinova’ genetic background led to the generation of M2117 transgenic line, who’s genomic DNA amplified gene-specific fragments, which ‘Pinova’ itself failed to amplify. It is logical that sequences of amplicons amplified by M2117 are the same as the 05210-147, which possesses a resistant phenotype to *E. amylovora* strain Ea222. However, it would be hasty to suggest or jump to the conclusion that M2117 will possess a resistant phenotype. For this, a phenotypic evaluation of this transgenic line is necessary.

Furthermore, the apple reference genome, GDDH13 [16] showed strong identity with the candidate gene sequence, as shown in this study. However, noteworthy is the 18 bp deletion and several other SNPs within the homolog gene sequence. It is in fact interesting that this indel also situates in the first exon of the homolog gene. The 18 bp deletion in GDDH13, which causes 15 amino acid difference in the protein level, is however potentially sufficient to affect the corresponding phenotypes, or render the homolog gene inactive or non-functional. One base pair switch in the *Pi-d2* gene was sufficient to distinguish resistance and susceptibility to rice blast [20] and a single nucleotide mutation in *E. amylovora* was sufficient to breakdown the fire blight resistance of *M*. ×*robusta* 5 [19]. Although, there is sequence identity between the gene and the homolog on GDDH13, the apple cultivar ‘Golden Delicious’ is highly susceptible to fire blight [12] whereas MAL0045—the source of *FB_Mfu10*, is highly resistant to different strains of the pathogen [15].

## Limitations

Although the aforementioned results confirm the results of Emeriewen et al. [13] and reinforces this receptor kinase gene as a strong fire blight candidate gene, the generation of a single transgenic line so far and the failure to report its phenotypic evaluation is a limitation of the study. This is because the line is still too small for phenotypic evaluation. Moreover, this study primarily aimed at characterizing the genomic sequences of this line and MAL0045-derived F1 progeny, to confirm the initial study [13]. A larger scale study to generate more transgenic lines is underway. Further studies will aim at generating more transgenic lines overexpressing this candidate gene in ‘Pinova’ as well as in ‘Gala’—another susceptible cultivar, and more importantly, evaluate whether it is functional. These experiments are in progress and we intend to publish the results regardless of the outcome. These results are highly anticipated as to date the only functionally proven fire blight resistance gene in *Malus* is *FB_MR5* [21], isolated from *M*. ×*robusta* 5 fire blight resistance locus region on LG3 [5, 11], and which is already broken down by highly virulent and mutant strains of *E. amylovora* [19].

## Supplementary Information

**Additional file 1: File S1.** Binary plasmid vector harbouring the candidate gene under its own promoter sequence used for transformation. STPK = serine/threonine protein kinase – candidate gene sequence.

**Additional file 2: File S2.** Alignment of *FB_Mfu10* candidate gene sequence against the GDDH13 genome. The significant 18 bp indel is highlighted in red. Two nucleotides ‘GA’ after position 3553 shown in this alignment completes *FB_Mfu10* sequence (i.e. 3555 bp).

**Additional file 3: File S3.** Predicted 865 amino acid from GDDH13 homolog. See Emeriewen et al. [13] for the predicted proteins of this candidate gene.

## Data Availability

Data generated from this study are published within this article. Sequences obtained in this study were aligned against the GDDH13 genome [16]. Further materials can be provided on request from the corresponding author, Ofere Francis Emeriewen.

## Abbreviations

aa: Amino acid
bp: Base pair
GDDH13: Golden delicious doubled haploid genome
indel: Insertion/deletion
BACs: Bacterial artificial chromosome
LG: Linkage group
ORF: Open reading frame
PCR: Polymerase chain reaction
RT-PCR: Reverse transcription PCR
ng: Nanogram
DNA: Deoxyribonucleic acid
nptII: Neomycin phosphotransferase
dNTPs: Deoxyribonucleotide triphosphate
min: Minutes
sec: Seconds
kb: Kilobase
