# Supplementary material for: Characterization of genomic DNA sequence of the candidate gene for FB_Mfu10 associated with fire blight resistance in Malus species
Source: BMC Res Notes. 2021 Jul 27;14:291. doi: 10.1186/s13104-021-05709-2 (PMC8314441; doi:10.1186/s13104-021-05709-2)
Supplement: Supplementary file 1 — Additional file 1: File S1. Binary plasmid vector harbouring the candidate gene under its own promoter sequence used for transformation. STPK = serine/threonine protein kinase – candidate gene sequence. [file 13104_2021_5709_MOESM1_ESM.docx]

**File S1**. Binary plasmid vector harbouring the candidate gene under its own promoter sequence used for transformation. STPK = Serine/threonine protein kinase – candidate gene sequence
