# Supplementary material for: Characterization of genomic DNA sequence of the candidate gene for FB_Mfu10 associated with fire blight resistance in Malus species
Source: BMC Res Notes. 2021 Jul 27;14:291. doi: 10.1186/s13104-021-05709-2 (PMC8314441; doi:10.1186/s13104-021-05709-2)
Supplement: Supplementary file 2 — Additional file 2: File S2. Alignment of FB_Mfu10 candidate gene sequence against the GDDH13 genome. The significant 18 bp indel is highlighted in red. Two nucleotides ‘GA’ after position 3553 shown in this alignment completes FB_Mfu10 sequence (i.e. 3555 bp). [file 13104_2021_5709_MOESM2_ESM.docx]

**File S2**. Alignment of *FB_Mfu10* candidate gene sequence against the GDDH13 genome. The significant 18 bp deleted in GDDH13 is highlighted in red. Two nucleotides ‘GA’ after position 3553 shown in this alignment completes the candidate gene sequence (i.e. 3555 bp).
