# Supplementary material for: Characterization of genomic DNA sequence of the candidate gene for FB_Mfu10 associated with fire blight resistance in Malus species
Source: BMC Res Notes. 2021 Jul 27;14:291. doi: 10.1186/s13104-021-05709-2 (PMC8314441; doi:10.1186/s13104-021-05709-2)
Supplement: Supplementary file 3 — Additional file 3: File S3. Predicted 865 amino acid from GDDH13 homolog. See Emeriewen et al. [13] for the predicted proteins of this candidate gene. [file 13104_2021_5709_MOESM3_ESM.docx]

MGSCRTCLLIFSSLVVWTYYYTAASAQVTHKPGDTLKPGESLNSTTWLCSAMGTFCLGFC

VYDKSNSSQLCIWARDTSNTGWIASRDKPVLYPTGVLTLDKNKTLKIMDQGRTRLELYSA

SRETANTNTSTVVATLLDSGNFILQEVNTIHGSKNRILWQSFDHPTDTLYPGMMLGVNHR

NGHMLSLTSWSSDYNPKPEPFTLEWDYKTQELQIKRRGVVYWTSGALTNKRFKLLRRRYN

FSIVSNKNEDYFFYYSLSQTSASEWYLTSSGLLFDYGGVDIARADNCYGYNTDGGCQRWA

EKPTCRHVGDIFELKTGFFKPTTTNSTPDSTFPSDSNESLSISDCKDSCWKNCECLGYTF

LNADDESGCQYYTGINWEFIQDFTGDSTQNFNMLKTKSPHSNGTKKRIFIGTGITVATLL

LMVPCIACYVVRRRKFALSGEKETNIIEDELLDLMRSDRPTDANARQNDGNMRHDLSVFS

YASVMAATCNFSQENKLGQGGFGPVYKGKLVTGQEVAVKRLSKCSGQGTLEFKNELILIY

ELQHKNLVKLFGFCIHGEERMLIYEYMPNKSLDYFLFDSTRVTLLDWKKRFSIIEGIAQG

LLYLHKYSRVTVIHRDLKASNILLDENMNPKISDFGMARIFKHNELEANTNRVVGTYGYM

SPEYAMEGLFSIKSDVYSFGVLMLEIVSGRRNNSFYNADRLLNIVGYAWELWKEGTVLEL

MDPALGDSCIKDQLLRCVHVGLLCVEENAADRPTMSDVVSMLTNQSSPLPLPAKAAFFTG

RNVVENAVVAVPVHIITVDVPMRKQLSELAAFFLRHKVLNQNVFSRSCGTPMEASVLRNT

RHVSTIPSSSYYIGVGHAIWLKEGR

**File S3**. Predicted 865 amino acid from GDDH13 homolog. See Emeriewen et al. 2018 for the predicted proteins of this candidate gene
